# Early postnatal antibiotic-associated gut microbiota alterations might promote long-term lipid metabolism via brown adipose tissue metabolic programming

**DOI:** 10.1080/19490976.2026.2665885

**Published:** 2026-05-04

**Authors:** Huijing Liang, Fengling Jiang, Lei Ren, Xiaoting Li, Simou Wu, Jinxing Li, Liang Li, Xiaolei Ze, Xi Shen, Fang He

**Affiliations:** a Department of Nutrition and Food Hygiene, West China School of Public Health and West China Fourth Hospital, Sichuan University, Chengdu, Sichuan, China; b BYHEALTH Institute of Nutrition & Health, Guangzhou, Guangdong, China; c Department of Infectious Disease Prevention, Sichuan Tianfu New Area Public Health Center, Chengdu, Sichuan, China

**Keywords:** Early postnatal, gut microbiota, antibiotic, lipid metabolism, metabolic programming, brown adipose tissue

## Abstract

The Developmental Origins of Health and Disease theory describes early life as a critical window for long-term metabolic health. Accumulating evidence has identified the gut microbiota as a key mediator of early-life metabolic programming. This study utilized antibiotic intervention in neonatal mice to investigate the long-term effects of early postnatal gut microbiota perturbations on adult lipid metabolism and examined the underlying mechanisms involving both thermogenic adipose tissue programming and microbiota structural remodeling. We found that early postnatal antibiotic exposure significantly disrupted the normal developmental assembly of the gut microbiota. Surprisingly, these alterations were associated with partial attenuation of high‑fat diet‑induced lipid metabolic disturbances in adulthood, an effect that was more pronounced in male mice than in female mice. Mechanistically, the observed metabolic improvement appeared to be associated with brown adipose tissue (BAT) thermogenic activation rather than with white adipose tissue browning or persistent gut microbiota restructuring. Early postnatal antibiotic exposure-associated gut microbiota alterations were linked to enhanced BAT development, potentially via interleukin-6 signaling and M2 macrophage polarization, suggestive of a metabolic programming effect that enhanced adaptive thermogenesis and improved long-term lipid homeostasis. These findings indicate that the gut microbiota might represent a modifiable factor influencing adipose tissue development, highlighting the potential of targeting the microbiota–BAT interplay in early life for obesity prevention strategies.

## 1. Introduction

Overweight and obesity are defined as abnormal or excessive fat accumulation that presents a health risk. Extensive evidence has established overweight/obesity as a major risk factor for various noncommunicable diseases, including cardiovascular disease, type 2 diabetes, nonalcoholic fatty liver disease, and osteoarthritis, which substantially compromise quality of life and increase mortality.^1^ According to the World Obesity Federation, the global prevalence rates of overweight and obesity in adults reached 38% and 14%, respectively, in 2020, with projections indicating increases to 51% and 24%, respectively, by 2035, representing an estimated 2.9% of global GDP in economic burden.^2^ These alarming trends underscore the urgent need for effective strategies to prevent and manage metabolic disorders.

The Developmental Origins of Health and Disease (DOHaD) hypothesis posits that early-life factors (e.g., malnutrition, overnutrition, pharmaceutical, and chemical exposure) can induce genomic imprinting that permanently “programs” organ structure and metabolism, thereby shaping long-term health trajectories and disease susceptibility.^3-5^ Epidemiological studies have established associations between adverse early-life events (e.g., abnormal birth weight, shortened breastfeeding duration) and increased obesity risk later in life,^6-8^ highlighting this developmental period as a critical window for obesity prevention. Notably, the early postnatal phase also represents a pivotal timeframe for gut microbiota establishment. Emerging evidence from the past decade has linked disrupted microbial colonization during this period with heightened risks of metabolic disorders, including overweight/obesity, type 2 diabetes, and cardiovascular diseases,^9^^,^^10^ suggesting that the gut microbiota represents a key mediator of early-life metabolic programming.^11^^,^^12^

Gut microbiota establishment during early life represents a dynamic and complex developmental process. From birth to approximately 3 y of age, infants undergo progressive microbial colonization, leading to an adult-like gut ecosystem.^13-15^ Prior to this maturation (particularly before weaning), the gut microbiota exhibits marked structural instability and low diversity, rendering it highly susceptible to external perturbations such as feeding practices, environmental exposures, and antibiotic administration. Notably, antibiotics are frequently used as experimental tools to disrupt microbial colonization, enabling investigation of the effects of early-life gut microbiota disturbances on long-term metabolic outcomes. For instance, a large Finnish cohort study identified antibiotic use before 6 months as a risk factor for overweight at 2 y old.^16^ Chen et al. demonstrated that antibiotic exposure during the first postnatal year significantly alters the gut microbiota composition and elevates childhood obesity risk.^17^ Blaser’s research group revealed that low-dose antibiotic exposure before weaning, but not after weaning, induces a growth promotion phenotype in mice, with fecal microbiota transplantation experiments confirming that this effect is mediated by microbial disruption rather than direct antibiotic action.^18^^,^^19^ Although these studies suggested that early-life antibiotic-induced gut microbiota disruption can elevate long-term metabolic risks, existing evidence is conflicting. Some studies reported no significant effect on body weight or obesity susceptibility ^20^^,^^21^ or even reduced weight following weaning-period antibiotic exposure.^22^ Furthermore, few existing studies have examined the continuous developmental trajectory from infancy to adult stages, highlighting the need for further investigation.

The mechanisms linking early-life gut microbiota perturbations to long-term metabolic outcomes remain poorly understood. Recent research has directed increasing attention toward the metabolic programming role of thermogenic adipose tissues. Brown adipose tissue (BAT), a classic thermogenic fat depot, contains abundant mitochondria and highly expresses uncoupling protein 1 (UCP1), enabling nonshivering thermogenesis. Additionally, white adipocytes can undergo “browning” in response to various stimuli (e.g., cold, exercise, hormonal signals), transforming into UCP1-expressing beige adipocytes that functionally resemble BAT. Subcutaneous white adipose tissue (WAT) exhibits significantly greater browning potential than visceral WAT.^23^ Various studies have demonstrated that UCP1-expressing adipose tissues promote energy expenditure via nonshivering thermogenesis, thereby improving systemic metabolism.^24-26^ The early postnatal period represents a critical window for adipose tissue development,^27^^,^^28^ with studies demonstrating an inverse correlation between neonatal BAT content and later adiposity.^29^ As this developmental phase coincides with gut microbiota establishment and considering the direct regulatory effects of gut microbes on BAT activity and WAT browning,^30^^,^^31^ we hypothesized that the early postnatal gut microbiota influences metabolic programming by modulating the development and function of thermogenic adipose tissue. However, direct evidence linking the postnatal gut microbiota to long-term metabolic outcomes via thermogenic fat programming remains elusive, warranting further investigation into these potential mechanisms.

Conversely, recent studies have revealed that antibiotic exposure can cause persistent, rather than transient, alterations in microbial composition and function.^32^^,^^33^ Because distinct gut bacterial species utilize different nutrient substrates, exhibit varied energy harvest capacities, and produce unique metabolites, they might directly influence host obesity susceptibility through these metabolic preferences.^34-36^ These findings indicate that the early-life gut microbiota might additionally program long-term metabolism through sustained modulation of microbial structure and function.

In this study, we hypothesized that disturbed gut microbiota colonization during the postnatal period might have long-term effects on lipid metabolic homeostasis in adulthood. We treated neonatal mice with antibiotics to perturb microbial establishment and longitudinally assessed lipid metabolic consequences in adulthood. The intermediate mechanism linking early-life gut microbiota to long-term lipid metabolism was explored from two pathways: thermogenic adipose tissue metabolic programming and gut microbiota structure metabolic programming.

## 2. Materials and methods

### 2.1. Mice and treatment

In total, 16 pregnant C57BL/6J mice (9 weeks old, gestational day 12–14) were obtained from GemPharmatech Co., Ltd. (Chengdu, China; License No. SCXK(Chuan)2020-034). Mice were housed in the specific pathogen-free–grade animal facility of West China School of Public Health, Sichuan University (License No. SYXK(Chuan)2023-0011) under controlled temperature (23 °C ± 1 °C), humidity (50%–70%), and light exposure (12-h/12-h light/dark cycle), with *ad libitum* access to food and water.

**Figure 1.**
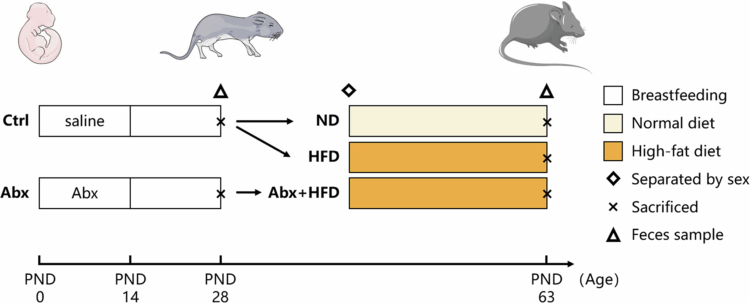
Experimental design. The experimental groups for final data analysis included: weaning mice at PND28 (Ctrl, *n* = 12; Abx, *n* = 12) and adult mice at PND63 (ND males, *n* = 12; HFD males, *n* = 12; Abx+HFD males, *n* = 12; ND females, *n* = 12; HFD females, *n* = 12; Abx+HFD females, *n* = 12).

After adaptive feeding, pregnant dams delivered naturally. As presented in Figure 1, the neonatal pups were randomly divided into the control (Ctrl, *n* = 60) and antibiotic groups (Abx, *n* = 36). All pups were nursed freely by the dams within the same litter. On postnatal days (PNDs) 0–14, pups in the Ctrl group received daily oral gavage of sterile saline, whereas those in the Abx group received ceftriaxone sodium (100 mg/kg·bw; Aladdin, Shanghai, China). On PND 28, 12 mice from each group were randomly selected, weighed, fasted for 8 h, and subsequently euthanized (the sex ratio was 1:1), whereas all remaining mice were weaned and separated by sex. The Ctrl group was randomly divided into a normal diet (ND, *n* = 12/sex) and a high-fat diet (HFD) groups (*n* = 12/sex). Mice in the ND group were fed standard chow daily (270 kcal/100 g; 10% of energy from fat, 20% from protein, and 70% from carbohydrates; Dashuo, Chengdu, China), whereas those in the HFD group received a HFD (521 kcal/100 g; 60% of energy from fat, 20% from protein, and 20% from carbohydrates; Research Diets, New Brunswick, NJ, USA). Meanwhile, 24 mice (*n* = 12/sex) in the Abx group were fed the HFD daily (Abx+HFD group). Body weight and food intake were measured every 3 days. On PND 63 (corresponding to murine adulthood), the experiment ended, and all remaining mice were weighed, fasted for 8 h, and euthanized.

On PNDs 28 and 63, fresh stool pellets from mice were collected, and rectal temperature was measured using a digital animal thermometer (KEW BASIS, Nanjing, China). Blood samples were collected and centrifuged to isolate sera. The liver, spleen, thymus, interscapular brown adipose tissue (iBAT), inguinal white adipose tissue (ingWAT), perigonadal WAT (pgWAT), mesenteric WAT (mWAT), and perirenal white adipose tissue (prWAT) of mice were isolated and used to calculate organ indices. Fecal or blood samples from two or three randomly selected mice within each group were pooled to form one composite biological replicate. These pooled samples were subsequently used for analyses, including 16S rRNA sequencing, fecal SCFA quantification, blood lipid measurement, and serum hormone and cytokine determination (excluding serum insulin).

To further examine the immediate effects of early postnatal antibiotic intervention on the gut microbiota and iBAT thermogenesis, a supplemental experiment was designed. Using neonatal mice and housing conditions identical to those in the main study, antibiotic administration at the same dosage was initiated from PND 0 and continued until PND 21 (PND 14 was not chosen because of the difficulty of obtaining sufficient tissue and fecal samples at that age). Fresh fecal samples were collected immediately prior to euthanasia, after which organ indices were measured and iBAT gene expression was analyzed.

All experimental procedures were officially approved by the Ethics Committee of West China Fourth Hospital and West China School of Public Health, Sichuan University (Approval No.: Gwll2022059) and conducted in accordance with the institutional guidelines for animal experimentation at West China School of Public Health.

### 2.2. Bacterial DNA extraction and 16S rRNA sequencing

Genomic DNA from fecal samples collected on PNDs 28 and 63 was extracted using the TIANamp Stool DNA Kit (Tiangen, Beijing, China) following the manufacturer's protocol. Using previously reported methods,^37^ the V3–V4 hypervariable regions of bacterial 16S rRNA genes were amplified and then sequenced on an Illumina NovaSeq6000 Instrument (Illumina, San Diego, CA, USA).

### 2.3. Bioinformatics

Sequencing data processing and bioinformatics analysis were performed according to previously reported methods.^38^ Briefly, sequencing data were filtered, and high-quality effective tags were clustered into operational taxonomic units (OTUs) with 97% similarity. The most abundant sequences within each OTU were selected as representative sequences. Taxonomic annotation was performed using the Mothur algorithm and SSU rRNA database (SILVA 138.1) with a threshold value of 0.8–1.0.^39^ The relative abundance of species was visualized using stacked bar plots generated using R software (v2.15.3). Representative sequences of OTUs were aligned using MUSCLE (v3.8.31) to establish phylogenetic relationships. Following data normalization, the processed sequences were subjected to downstream diversity analyses.

QIIME was used to calculate *α*-diversity indices (Chao1, Ace, Shannon, and Simpson). Beta diversity was assessed through weighted UniFrac distance matrices, followed by principal coordinates analysis (PCoA) to visualize intergroup variations. The Kruskal–Wallis test was used to analyze the differences between groups.

To identify statistically significant biomarkers between groups, we performed linear discriminant analysis (LDA) and effect size (LEfSe) using LEfSe software. All differentially abundant and biologically relevant features were ranked by effect size after LDA. An effect size threshold of >4 (on a log10 scale) was used for all biomarkers.

Functional prediction of gut microbiota was performed using Tax4Fun analysis as described by Asshauer et al.^40^ Briefly, prokaryotic whole-genome 16S rRNA sequences from the KEGG database were aligned against the SILVA SSU Ref NR database via the BLASTN algorithm to establish a correlation matrix, enabling functional annotation based on SILVA taxonomy. All predicted functional pathways at KEGG Level 2 were selected for comparative analysis, with relative abundance visualized through clustering heatmaps to illustrate intergroup differences in microbial functional profiles.

### 2.4. Fecal SCFA detection

SCFA concentrations in mouse fecal samples were quantified as described by Wu et al.^41^ Briefly, standard curves for acetic, propionic, butyric, isobutyric, valeric, isovaleric, and caproic acids (all from Sigma–Aldrich, St Louis, MO, USA) were generated to enable quantitative analysis. Fecal samples (50 mg each) were acidified with 15% phosphoric acid and then mixed with internal standard solution (isocaproic acid) and ether. Homogenized fecal samples were centrifuged at 12,000 rpm for 10 min at 4 °C to obtain supernatants. Then, 1 μL of each supernatant was automatically injected into a TRACE 1300 gas chromatography system (Thermo Fisher Scientific, Waltham, MA, USA) equipped with an Agilent HP-INNOWAX column (30 m, 0.25 mm ID, 0.25 μm) and detected by an ISQ 7000 mass spectrometer (Thermo Fisher Scientific).

### 2.5. Serum and liver lipids

Blood samples were centrifuged at 2000 × g at 4 °C to obtain serum, which was subsequently analyzed to determine total triglyceride (TG) and total cholesterol (TC) levels using a Chemray 800 automated biochemistry analyzer (Rayto, Shenzhen, China) according to the manufacturer’s protocols for the respective assay kits (Rayto).

Liver tissues (50 mg each) were homogenized in ice-cold tissue lysis buffer and centrifuged to obtain supernatants. Total protein concentrations were determined by the BCA assay using bovine serum albumin standards (APPLYGEN, Beijing, China) and normalized to equal concentrations. TG and TC levels in the supernatants were then quantified using commercial assay kits (APPLYGEN) by a Chemray 800 automated biochemistry analyzer.

### 2.6. Fasting blood glucose (FBG) measurements and oral glucose tolerance test (OGTT)

FBG measurements and OGTT were performed in all mice before euthanasia. After fasting for 8 h, FBG levels (0 min) in tail vein blood were measured using an Accu-Chek Active glucose meter (Roche, Mannheim, Germany). Thereafter, mice were gavaged with a 2.0 g/kg·bw glucose solution, and blood glucose levels were measured at 30, 60, 90, and 120 min after glucose gavage.

### 2.7. Enzyme-linked immunosorbent assay (ELISA)

Serum insulin levels were assessed using a commercial ELISA kit following the manufacturer’s instructions (Elabscience, Wuhan, China). The homeostasis model assessment of insulin resistance (HOMA-IR) index was calculated as (FBG × fasting insulin)/22.5. Serum peptide YY (PYY) levels were also analyzed using an ELISA kit (Novus Biologicals, Littleton, CO, USA). Serum leptin, interleukin (IL)-2, IL-5, IL-6, and tumor necrosis factor (TNF)-*α* levels were determined using a Luminex assay (R&D Systems, Minneapolis, MN, USA) and a Luminex 200 multiplexing instrument (Merck Millipore, Burlington, MA, USA). Assays were performed according to the manufacturer’s instructions.

### 2.8. Histopathology

Fresh iBAT and ingWAT samples were dissected and immediately fixed in 10% neutral phosphate-buffered saline formalin for 72 h. Following fixation, tissues were processed through standard paraffin embedding, sectioned, and stained with hematoxylin and eosin (H&E). Histological examination was performed using a light microscope (Olympus, Tokyo, Japan), with three random fields selected per slide. Adipocyte cross-sectional areas were quantified using ImageJ software, with the mean value from three fields representing the sample's adipocyte size.

Additionally, immunofluorescence staining was performed to detect UCP1 expression in iBAT and ingWAT sections. After antigen retrieval and blocking, tissue sections were incubated with primary anti-UCP1 antibody (1:100; Cell Signaling, Beverly, MA, USA) overnight at 4 °C, followed by secondary antibody (1:500; Thermo Fisher Scientific) for 45 min at room temperature. Nuclei were counterstained with DAPI (1:500; Solarbio, Beijing, China). Fluorescence images were acquired using a BX53 epifluorescence microscope (Olympus). UCP1 signal intensity was quantified using QUPATH software, with three randomly selected fields per sample analyzed for mean fluorescence intensity.

Eight liver tissues were randomly selected from each group for oil red O staining. Fresh liver tissues were fixed, embedded in OCT compound (Sakura Finetek, Tokyo, Japan), cryosectioned, and stained with oil red O (Solarbio). Immediate microscopic assessment was performed to evaluate hepatic lipid accumulation. Oil red O-stained areas were analyzed using QuPath software. The percentage stained area relative to the total tissue area was calculated for each field, with the mean value from three fields representing the lipid droplet ratio (%) for each specimen.

### 2.9. RNA isolation and RT-qPCR

Total RNA was extracted from iBAT, ingWAT, and proximal jejunum samples (20 mg each) using an Animal Total RNA Isolation Kit (Foregene, Chengdu, China), according to the manufacturer’s instructions. Total RNA was reverse-transcribed into cDNA using the iScript cDNA Synthesis Kit (Bio-Rad, Hercules, CA, USA). The 10-μL reaction mixture contained 2 μL of 5× iScript Reaction Mix, 0.5 μL of iScript Reverse Transcriptase, 1 μg of total RNA, and 6.5 μL of nuclease-free water. The cycling conditions were as follows: 5 min at 25 °C, 20 min at 46 °C, and 1 min at 95 °C for enzyme inactivation, followed by a 4 °C hold (C1000 Touch Thermal Cycler, Bio-Rad). The cDNA concentration was quantified and diluted to an appropriate concentration for qPCR.

qPCR of cDNA was then performed using SsoFast EvaGreen Supermix (Bio-Rad) on a CFX96 system (Bio-Rad) according to the manufacturer’s protocol. Each 10-μL reaction mixture contained 5 μL of 2× SYBR Green Supermix, 0.5 μL each of forward and reverse primers, 100 ng of cDNA template, and 2 μL of nuclease-free water. The amplification protocol consisted of initial denaturation at 98 °C for 30 s, followed by 40 cycles of denaturation at 98 °C for 15 s and annealing/extension at 60 °C for 30 s, with fluorescence acquisition at the end of each cycle. Melting curve analysis was performed to verify amplification specificity. All primers used in this study were designed using the National Center for Biotechnology Information database and synthesized by Sangon Biotech (Shanghai, China), with sequences listed in Table S1. The relative expression of all target genes was calculated using *Gapdh* or *β-actin* as the internal reference gene, with normalization performed according to the 2^−ΔΔCt^ method. Supplementary Table S2 presents raw Ct values of the internal reference gene across all experimental groups and samples.

### 2.10. Statistical analysis

All statistical analyses were performed using SPSS 25.0 software unless otherwise specified. Continuous variables were expressed as the mean ± standard deviation (SD). For two-group comparisons, Student’s *t*-test was applied for normally distributed data, whereas Wilcoxon’s rank-sum test was used for non-normally distributed data. Multiple-group comparisons were performed using one-way ANOVA followed by the LSD test or Games–Howell test. The Kruskal–Wallis test was employed for non-normally distributed multi-group data. All tests were two-tailed, with *p* < 0.05 considered statistically significant.

## 3. Results

### 3.1. Short-term effects of early postnatal antibiotic exposure on gut microbiota

**Figure 2.**
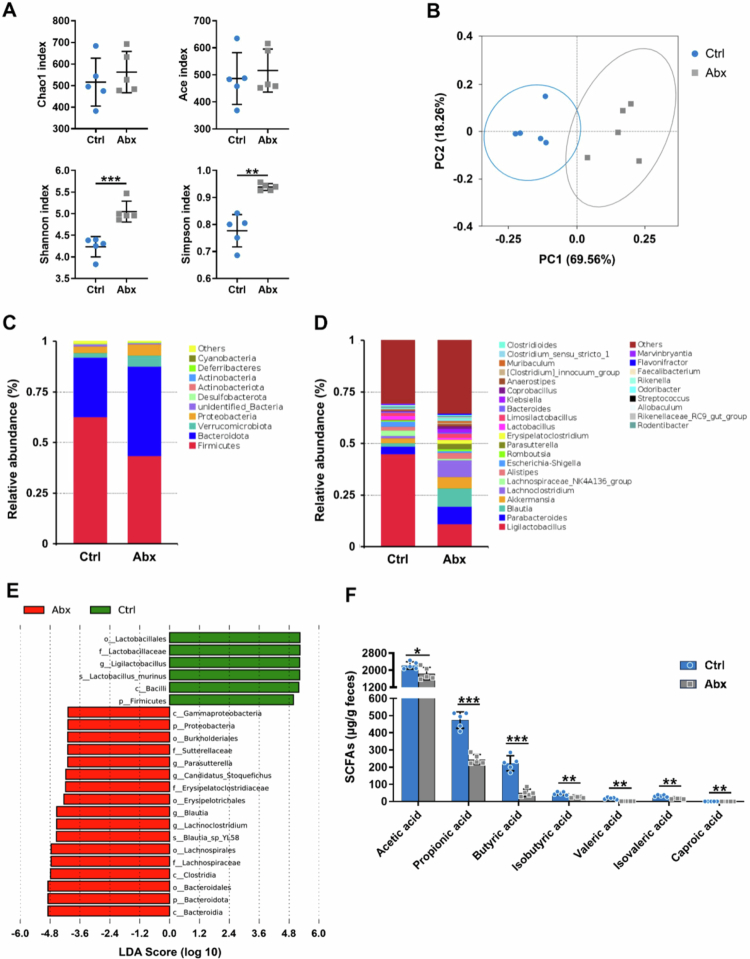
Changes of gut microbiota and SCFA levels in weaning mice (PND 28). (A) The α-diversity of fecal microbiota. (B) PCoA of the fecal microbiota. (C) The fecal microbiota composition at the phylum level. (D) The fecal microbiota composition at the genus level. (E) LDA score of the fecal microbiota. (F) Fecal SCFA levels in the mice. The data are presented as the mean ± SD (*n* = 5). ^*^p < 0.05, ^**^p < 0.01, ^***^p < 0.001.

In this study, we first examined the short-term effects of early postnatal antibiotic exposure on gut microbiota diversity and composition in weaning mice (PND 28). As presented in Figure 2A, after a 2-week natural recovery period, neonatal antibiotic exposure resulted in increased gut microbial diversity compared with that in the Ctrl group, with both the Shannon (*p* < 0.001) and Simpson indices (*p* < 0.01) revealing marked elevation. The observed increase in *α*-diversity might be attributable to the clearance effect of antibiotics, creating ecological niches that facilitate the expansion of nondominant bacterial groups during the recovery period. As presented in Figure 2B, PCoA based on the weighted UniFrac distance revealed a clear separation between the two groups along the first principal component (PC1), and AMOVA testing confirmed significant structural differences in bacterial communities (*p* < 0.01). These findings demonstrate that early postnatal antibiotic exposure exerts profound effects on the gut microbiota of weaning mice.

Further analysis of fecal bacterial community composition revealed that at the phylum level (Figure 2C); the gut microbiota of weaning mice was predominantly composed of Firmicutes, Bacteroidota, Verrucomicrobiota, and Proteobacteria. Compared with that in the Ctrl group, the relative abundance of Firmicutes was significantly decreased in the Abx group (43.54% vs. 62.64%), whereas that of Bacteroidota (44.11% vs. 29.43%) and Proteobacteria (5.54% vs. 3.11%) was markedly increased. At the genus level (Figure 2D), *Ligilactobacillus* dominated the fecal microbiota in Ctrl mice, whereas Abx mice exhibited a more even distribution of bacterial taxa, consistent with the increased Shannon and Simpson indices. Comparing with that in the Ctrl group, the relative abundance of *Ligilactobacillus* (10.93% vs. 45.06%) was significantly decreased in the Abx group, whereas that of *Lachnoclostridium* (8.22% vs. 1.36%), *Blautia* (8.77% vs. 2.38%), *Parasutterella* (2.82% vs. 0.27%), *Klebsiella* (1.67% vs. 0.12%), and *Anaerostipes* (1.04% vs. 0.28%) was significantly increased.

To identify statistically and biologically relevant biomarker taxa, we performed LEfSe with a focus on lower taxonomic levels. As presented in Figure 2E, the fecal microbiota of Ctrl mice was primarily characterized by *Ligilactobacillus* and its descendant species *Lactobacillus murinus*, whereas the signature taxa in the Abx group included *Lachnoclostridium*, *Blautia*, *Candidatus Stoqefichus*, and *Parabacteroides*.

Given the importance of bacterial metabolites in lipid metabolic functions, we measured SCFA production alongside microbial profiling. As presented in Figure 2F, the Abx group exhibited significantly diminished production of all detected SCFAs (acetic, propionic, butyric, isobutyric, valeric, isovaleric, and caproic acids; all *p* < 0.05) compared with that in the Ctrl group, indicating a profound impairment of microbial metabolic function.

### 3.2. Early postnatal antibiotic-associated gut microbiota alterations are linked with alleviated HFD-induced metabolic dysfunction in male adult mice

We further examined the effects of postnatal antibiotic-associated gut microbiota alterations on long-term lipid metabolic responses to HFD feeding. Considering the pronounced sex differences in energy metabolism after weaning, all results from adult mice were analyzed separately by sex.

**Figure 3.**
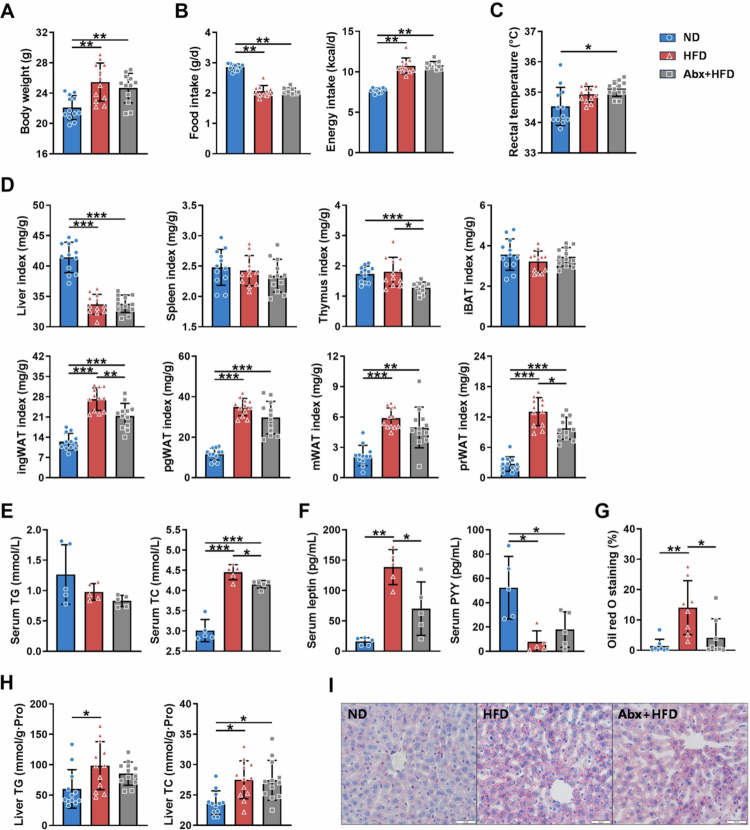
Lipid metabolic phenotypes in adult male mice (PND 63). (A) Body weight of mice at PND 63 (*n* = 12). (B) Average daily food intake and energy intake after weaning (*n* = 12). (C) Rectal temperature (*n* = 12). (D) Organ indices and adipose tissue indices (*n* = 12). (E) Serum lipid levels (*n* = 5). (F) Serum metabolic hormone levels (*n* = 5). (G) Quantification of oil red O (*n* = 8). (H) Liver lipid levels (*n* = 12). (I) Representative images of oil red O-stained liver sections. Scale bar, 20 μm. The data are presented as the mean ± SD. ^*^p < 0.05, ^**^p < 0.01, ^***^p < 0.001.

In adult male mice (PND 63), HFD feeding resulted in profound lipid and glucose metabolic disturbances relative to the findings in ND controls, including significant elevations in body weight, subcutaneous fat (ingWAT), visceral fat (pgWAT, mWAT, prWAT), serum TC and leptin, hepatic lipids, and FBG and increases in HOMA-IR (Figures 3, S4). Unexpectedly, with equivalent caloric intake (Figure 3B), early postnatal antibiotic exposure attenuated, rather than exacerbated, HFD-induced lipid metabolic disorders. Compared with the findings in the HFD group, the Abx + HFD group exhibited significantly decreased ingWAT (*p* < 0.01) and prWAT (*p* < 0.05), serum TC (*p* < 0.05) and leptin (*p* < 0.05) levels, and hepatic steatosis (*p* < 0.05) and trends toward reductions in serum and liver TG levels. Notably, although the absolute rectal temperature of the ND group indicated a hypothermic state, the significant elevation in temperature in the Abx+HFD group suggested a relative increase in thermogenic output (*p* < 0.05; Figure 3C). These results suggest a potential mechanistic link between improved lipid metabolism and enhanced thermogenesis, which requires further exploration.

As presented in Figures S2 and S3, compared with the findings in male mice, female mice exhibited less pronounced lipid and glucose metabolic disturbances in response to HFD feeding. The female cohort exhibited lower food intake, smaller gains in body weight and fat mass, and greater inter-individual variability. Compared with the results in the HFD group, the Abx+HFD group exhibited significantly decreased liver TC levels (*p* < 0.05; Figure S2H) and trends toward reductions in the prWAT index (*p* = 0.065; Figure S2D) and iBAT adipocyte size (Figure S3). However, many other comparisons in female mice did not reach statistical significance, whereas male mice exhibited a clearer difference between the HFD and Abx+HFD groups regarding WAT indices, serum lipid and leptin levels, and hepatic steatosis (Figure 3). These results suggest that under these experimental conditions, sex modifies the association between early antibiotic intervention and adult metabolic outcomes. Therefore, the subsequent mechanistic studies focus only on male mice to reduce variance.

### 3.3. Adaptive thermogenesis activation occurs in iBAT, but not in ingWAT, during adulthood

To investigate whether antibiotic-associated gut microbiota alterations activate thermogenesis, the subsequent study focused on iBAT and ingWAT because of their reported browning and thermogenesis potential. As female mice displayed less pronounced metabolic responses in preliminary analyses, all mechanistic studies were conducted exclusively in male animals.

**Figure 4.**
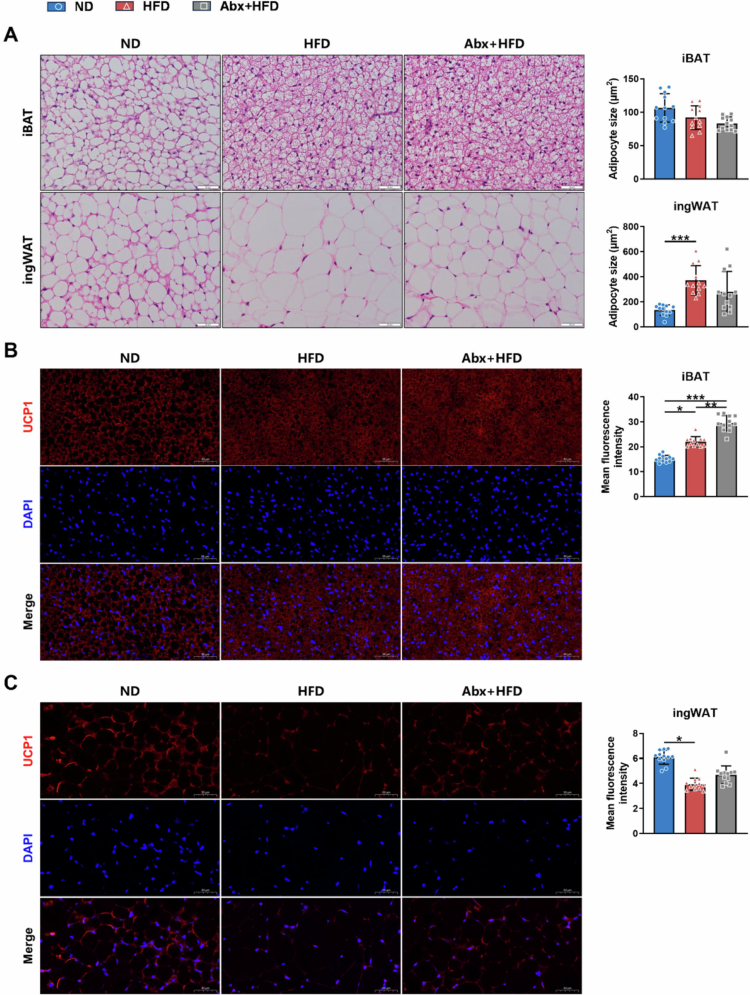
Morphology and UCP1 expressions in the iBAT and ingWAT of adult male mice (PND 63). (A) H&E staining on sections of iBAT and ingWAT of mice and quantification of adipocyte size. Scale bar, 20 μm. Immunofluorescent staining on sections of (B) iBAT and (C) ingWAT in mice and quantification of UCP1 fluorescence intensity. Scale bar, 50 μm. The data are presented as the mean ± SD (*n* = 12). ^*^p < 0.05, ^**^p < 0.01, ^***^p < 0.001.

As presented in Figure 4A, H&E staining revealed distinct morphological changes in both iBAT and ingWAT. HFD induced multilocular lipid droplets in iBAT, a phenotype particularly evident in the Abx+HFD group, which exhibited the most distinct brown adipocyte morphology and the smallest adipocyte area, albeit not reaching statistical significance. Conversely, ingWAT in the HFD group displayed substantial lipid deposition and significantly enlarged adipocytes compared with the findings in the ND group (*p* < 0.001). The Abx+HFD group exhibited a nonsignificant tendency for smaller ingWAT adipocytes than the HFD group.

Immunofluorescence staining revealed distinct UCP1 expression patterns between adipose depots (Figure 4B, C). In iBAT, the HFD group had significantly enhanced UCP1 fluorescence intensity versus that in the ND group (*p* < 0.05), with further elevation observed in the Abx+HFD group (*p* < 0.01 vs. the HFD group). Conversely, ingWAT exhibited markedly reduced UCP1 expression following HFD feeding (*p* < 0.05), which remained unaltered by postnatal antibiotic intervention. These results indicate selective activation of adaptive thermogenesis in iBAT, but not in ingWAT, during adulthood.

**Figure 5.**
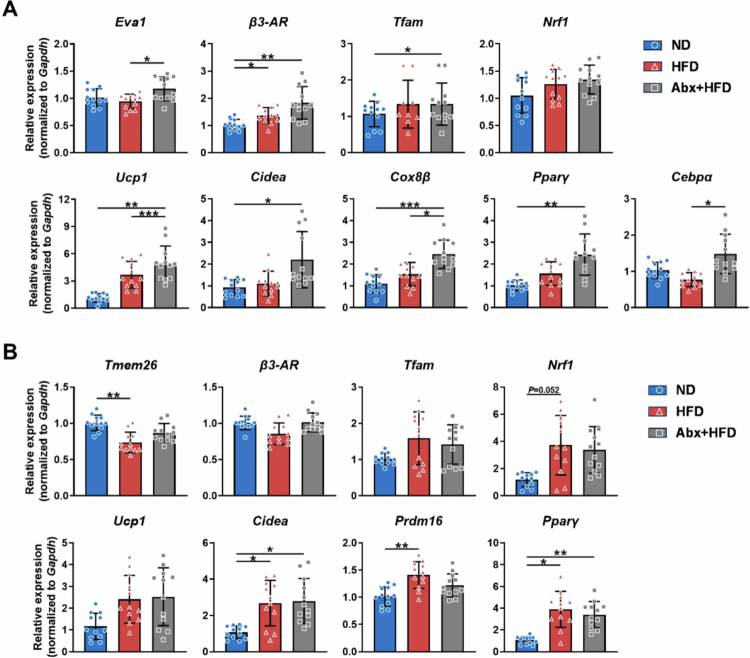
Thermogenic gene expression profiles in iBAT and ingWAT in adult male mice (PND 63). (A) Relative mRNA expression in mouse iBAT. (B) Relative mRNA expressions in the ingWAT of mice. Relative mRNA expression was normalized to that of the housekeeping gene *Gapdh*. The data are presented as the mean ± SD (*n* = 12). ^*^p < 0.05, ^**^p < 0.01, ^***^p < 0.001.

To further characterize thermogenic activation, we analyzed the transcriptional regulation of thermogenesis-related genes in both adipose tissues. In iBAT (Figure 5A), mice in the HFD group displayed significant upregulation of the classical thermogenic receptor (*β3-AR*, *p* < 0.05), as well as nonsignificant upward trends in the expression of thermogenic genes (*Ucp1*, *Cox8β*, *Cidea*, *Pparγ*) and mitochondrial function genes (*Tfam*, *Nrf1*), compared with the findings in the ND group, whereas the expression of the brown adipocyte marker (*Eva1*) remained unchanged. Compared with the findings in the HFD group, the Abx + HFD group exhibited significantly elevated mRNA expression of the brown adipocyte marker (*Eva1*, *p* < 0.05) and further enhanced thermogenic gene expression (*Ucp1*, *p* < 0.001; *Cox8β* and *Cebpα*, both *p* < 0.05). These results indicate that HFD feeding can stimulate adaptive thermogenesis in iBAT, whereas early postnatal antibiotic-associated gut microbiota alterations might lead to additional amplification of this adaptive response in adult male mice.

As presented in Figure 5B, HFD feeding also appeared to increase adaptive thermogenesis in ingWAT, manifested as a significant upregulation of thermogenic-related genes (*Cidea*, *Prdm16*, *PPARγ*, all *p* < 0.05) and a trend of upregulation of mitochondrial function genes (*Tfam*, *Nrf1*); however, HFD feeding simultaneously suppressed beige adipocyte markers (*Tmem26*, *p* < 0.01), indicating discordant browning responses. Notably, the Abx+HFD group exhibited similar expression patterns as the HFD group, confirming that early postnatal antibiotic-associated gut microbiota alterations promoted thermogenic activation specifically in iBAT, but not in ingWAT, during adulthood.

### 3.4. BAT metabolic programming is potentially linked with early-life gut microbiota alterations and sustained lipid metabolic outcomes

**Figure 6.**
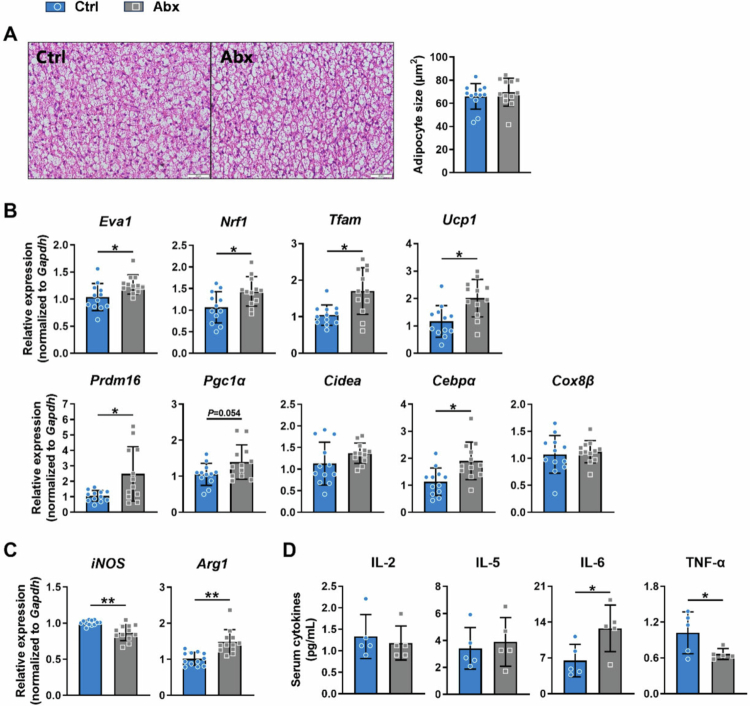
Developmental characteristics of iBAT and immune status in weaning mice (PND 28). (A) H&E staining on sections of iBAT from weaning mice and quantification of adipocyte size (*n* = 12). Scale bar, 20 μm. (B) Thermogenic gene expression profiles in iBAT (*n* = 12). (C) Relative mRNA expression of macrophage polarization markers in iBAT (*n* = 12). (D) Serum cytokine levels in weaning mice (*n* = 5). Relative mRNA expression was normalized to that of the housekeeping gene *Gapdh*. Data are presented as the mean ± SD. ^*^p < 0.05, ^**^p < 0.01, ^***^p < 0.001.

To investigate the potential metabolic programming effect in BAT, we further examined the development and function of iBAT in weaning mice (PND 28). As presented in Figures 6B and S1B, early postnatal antibiotic-associated gut microbiota alterations slightly increased rectal temperature at weaning (*p* = 0.060) and significantly increased the mRNA expression of brown adipocyte markers (*Eva1*), thermogenic genes (*Ucp1*, *Prdm16*, *Cebpα*), and mitochondrial function genes (*Tfam*, *Nrf1*) in iBAT (all *p* < 0.05), demonstrating enhanced thermogenic capacity during this critical developmental period. Furthermore, weaning mice in the Abx group exhibited significantly reduced mRNA expression of the M1 macrophage marker *iNOS* (*p* < 0.01) and elevated expression of the M2 marker *Arg1* (*p* < 0.01) in iBAT, suggesting immune regulation (Figure 6C). Serum cytokine analysis revealed increased IL-6 (*p* < 0.05) and decreased TNF-α (*p* < 0.05) levels in the Abx group versus the Ctrl group (Figure 6D). Collectively, these findings suggest that early postnatal antibiotic-associated gut microbiota alterations might be correlated with enhanced iBAT development, which promotes adaptive thermogenesis and programs long-term lipid metabolism.

**Figure 7.**
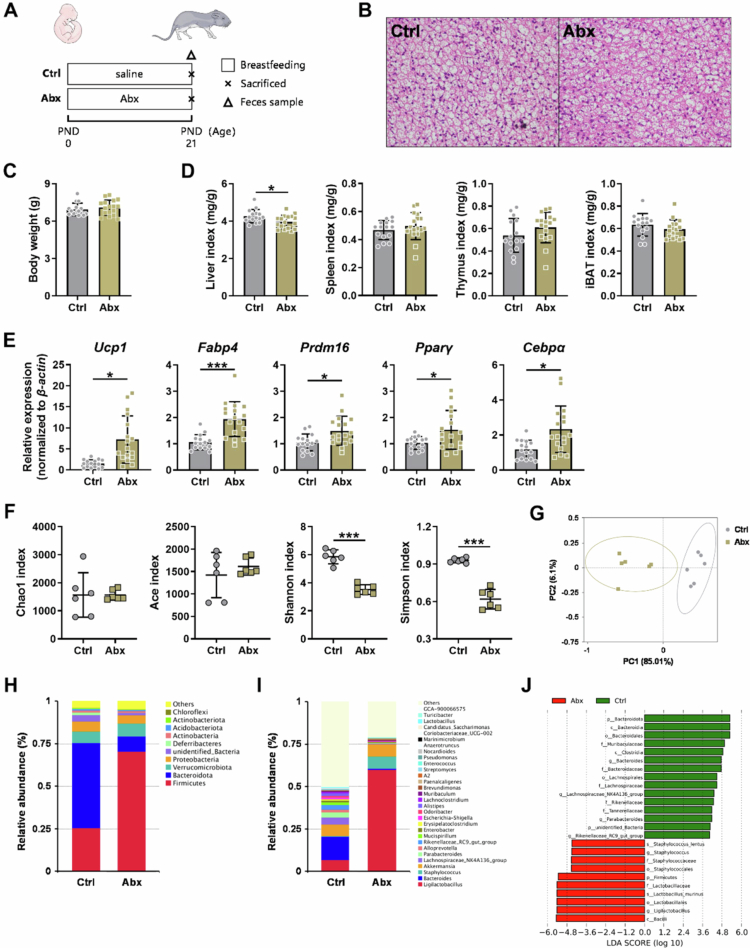
Supplemental experiments examining the immediate effects of early postnatal antibiotic exposure (PND 21). (A) Supplemental experimental design (Ctrl, *n* = 15; Abx, *n* = 17). (B) H&E staining of iBAT sections. Scale bar, 20 μm. (C) Body weight at PND 21 (*n* = 15–17). (D) Organ indices (*n* = 15–17). (E) Thermogenic gene expression profiles in iBAT (*n* = 15–17). (F) The α-diversity of fecal microbiota (*n* = 6). (G) PCoA of the fecal microbiota (*n* = 6). (H) The fecal microbiota composition at the phylum level (*n* = 6). (I) The fecal microbiota composition at the genus level (*n* = 6). (J) LDA score of the fecal microbiota (*n* = 6). Relative mRNA expression was normalized to that of the housekeeping gene *β-actin*. Data are presented as the mean ± SD. ^*^p < 0.05, ^**^p < 0.01, ^***^p < 0.001.

In this study, we assessed iBAT function after a 2‑week antibiotic intervention followed by a 2‑week natural recovery period. To directly examine the immediate effects of antibiotic-associated gut microbiota alterations on iBAT, a supplemental experiment was designed (Figure 7A), in which mice received continuous antibiotics from PND 0 to PND 21. Results from this experiment demonstrated that, at the end of the antibiotic intervention, the mRNA expression of adaptive thermogenesis‑related genes in iBAT (*Ucp1*, *Fabp4*, *Prdm16*, *Pparγ*, *Cebpα*) was significantly elevated (all *p* < 0.05; Figure 7E), verifying that early postnatal antibiotic‑associated gut microbiota alterations activated the thermogenic function of iBAT. In addition, the gut microbiota profile at PND 21 differed from that observed after the recovery period. Immediately after antibiotic cessation, both the Shannon and Simpson indices were significantly reduced (both *p* < 0.001; Figure 7F). PCoA revealed a distinct separation in community structure (AMOVA testing based on the weighted UniFrac distance, *p* = 0.001; Figure 7G). Compared with the findings in the Ctrl group, the Abx group exhibited significantly higher abundance of Firmicutes and *Ligilactobacillus* (both *p* < 0.05), whereas that of both Bacteroidota and *Lachnoclostridium* was significantly reduced (*p* < 0.05; Figure 7H–I). Together, the gut microbiota results from this supplemental experiment (Figure 7), and those obtained at weaning (Figure 2) allowed us to partially trace the dynamic changes in the gut microbiota induced by early‑postnatal antibiotic intervention.

### 3.5. No evidence was found for the gut microbiota structure metabolic programming mechanism

**Figure 8.**
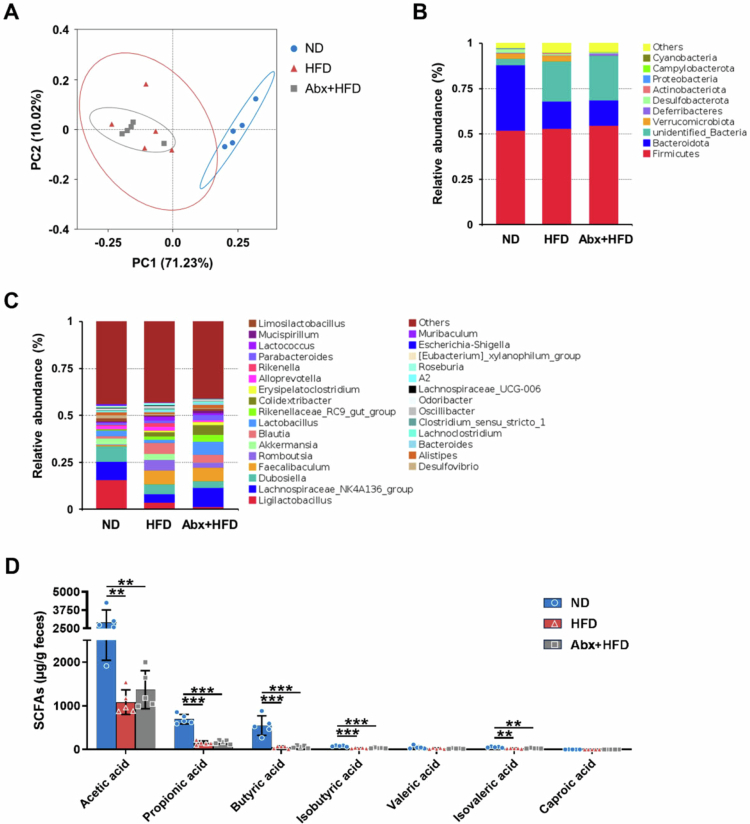
Changes in the gut microbiota and SCFA levels of adult male mice (PND 63). (A) PCoA of fecal microbiota. (B) The fecal microbiota composition at the phylum level. (C) The fecal microbiota composition at the genus level. (D) Fecal SCFA levels in adult male mice. Data are presented as the mean ± SD (*n* = 5). ^*^p < 0.05, ^**^p < 0.01, ^***^p < 0.001.

The potential microbiota structure metabolic programming mechanism was also explored (Figure 8). In adult male mice (PND 63), PCoA and AMOVA testing revealed significant structural differences between the ND group and both HFD-fed groups (*p* < 0.01), whereas the HFD and Abx+HFD groups formed overlapping clusters (*p* > 0.05). These findings demonstrate that HFD, rather than early postnatal antibiotic exposure, was the primary determinant of long-term gut microbiota composition. Similar to the microbial composition changes, SCFA profiles indicated that HFD significantly reduced the concentrations of nearly all tested SCFAs in adult male mice; however, no significant differences were observed between the HFD and Abx+HFD groups (Figure 8D).

**Figure 9.**
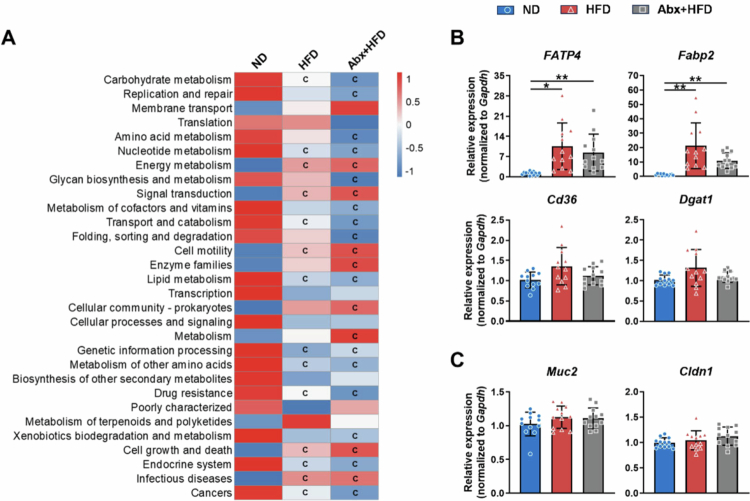
Gut microbiota functional prediction and jejunal gene expression in adult male mice (PND 63). (A) Functional pathways at KEGG Level 2 were selected for comparative analysis between the groups (*n* = 5). ^C^*p* < 0.05, compared with the ND group. (B) Fatty acid absorption and transport gene expression and (C) intestinal barrier function gene expressions in the proximal jejunum of adult mice (*n* = 12). Relative mRNA expression was normalized to that of the housekeeping gene *Gapdh*. Data are presented as the mean ± SD. ^*^p < 0.05, ^**^p < 0.01, ^***^p < 0.001.

Tax4Fun functional prediction analysis of gut microbiota at KEGG Level 2 (Figure 9A) revealed significant enrichment of multiple metabolic pathways in adult male mice in the Ctrl group, including carbohydrate metabolism, nucleotide metabolism, transport and catabolism, lipid metabolism, genetic information processing, amino acid metabolism, and the endocrine system. Although HFD feeding significantly suppressed the majority of these functional pathways (*p* < 0.05), the Abx+HFD group exhibited parallel functional alterations to the HFD group, with no significant differences observed between these two groups for any predicted function (*p* > 0.05). Furthermore, we analyzed jejunal gene expression profiles in adult male mice (Figure 9B). Both the HFD and Abx+HFD groups exhibited significantly greater expression of fatty acid absorption and transport genes (*FATP4*, *Fabp2*) than the ND group. However, no differences were observed between the HFD and Abx+HFD groups regarding the expression of genes related to fatty acid absorption or intestinal barrier function. These findings suggest that gut microbiota structural and functional alterations do not contribute to the improved metabolic phenotype observed in Abx+HFD mice. The results conclusively indicate that the metabolic benefits observed in Abx+HFD mice are not primarily driven by long-term changes in gut microbiota composition or function.

## 4. Discussion

In this study, ceftriaxone was selected to perturb the gut microbiota during the early postnatal period because of its broad‑spectrum activity, minimal intestinal absorption, and common clinical use in pediatrics as a third‑generation β‑lactam antibiotic. These characteristics ensure that its bactericidal effects remain localized to the gastrointestinal tract without systemic exposure, making it particularly suitable for establishing gut microbiota perturbation models and providing relevant insights for clinical consideration.^42^ In this study, we used this antibiotic to investigate the influence of transient neonatal microbial alteration on long-term lipid metabolism and elucidate the underlying mechanisms.

By integrating the 16S rRNA sequencing results from the main experiment (Figure 2; PND 28) and the supplemental experiment (Figure 7; PND 21), we could partially trace the dynamic changes in the gut microbiota of neonatal mice following antibiotic exposure. Antibiotic intervention disrupted a large number of low-abundance bacterial taxa and opened ecological niches, allowing *Ligilactobacillus* to become the dominant genus, which led to a significant decline in microbial diversity at PND 21. During the subsequent antibiotic-free recovery period, previously suppressed bacteria proliferated extensively, resulting in increased abundance of non‑dominant species (e.g., *Blautia*, *Alistipes*) and potentially pathogenic taxa such as *Proteobacteria*, whereas the colonization advantage of *Ligilactobacillus* diminished. These shifts collectively contributed to the observed elevation in α‑diversity at weaning. Thus, the eventual increase in gut microbiota diversity appears to stem from antibiotic‑induced disruption of early colonization patterns followed by recolonization rather than from a simple enrichment of health-promoting species. Coupled with the significant reduction in SCFA levels, these findings suggest that early postnatal antibiotic exposure exerts a negative effect on the assembly of the gut microbial ecosystem. These results underscore the importance of sampling timing after antibiotic intervention for interpreting microbiota outcomes, highlighting the need to consider multiple contextual factors when drawing conclusions about gut microbial changes.

After weaning, all of the tested mice were separated by sex and fed an HFD to induce lipid metabolic dysfunction. In previous studies, male C57BL/6J mice were most commonly used to establish metabolic disorders, whereas studies on female mice are insufficient.^43^^,^^44^ In the present study, following HFD feeding until early adulthood (9 weeks of age), both male and female mice developed characteristic metabolic disturbances, including increased body weight, excessive WAT accumulation, hepatic steatosis, and dyslipidemia. Notably, sex-specific differences were observed, with male mice exhibiting greater sensitivity to HFD, higher food intake and energy consumption, more pronounced increases in body weight and fat mass, lower inter-individual variability, and generally more severe metabolic phenotypes than female mice. These findings are consistent with previous reports^44^^,^^45^ indicating that male mice are more susceptible to HFD‑induced obesity and lipid metabolic disturbances than female mice and suggest that sex differences in metabolic responses might be attributable to variations in sex chromosomes, gonadal hormones, estrous cycles, physical activity, and leptin resistance levels.^44^^,^^46-48^ Our study found that male mice more readily develop leptin resistance than female mice, indicating weaker hormonal sensitivity and higher obesity predisposition. However, this work did not explore additional contributing factors, such as genetic influences, sex hormone fluctuations, or activity levels.

As well-established disruptors of gut microbiota homeostasis, antibiotics have been widely employed to study the consequences of early-life dysbiosis on metabolic health. Accumulating evidence supports a positive association, as demonstrated by Saari et al., who identified infant antibiotic exposure as a risk factor for overweight at 2 y of age.^16^ Similarly, Chen et al. reported that antibiotic use during infancy is correlated with elevated BMI in childhood, particularly among boys.^17^ In contrast to some previous findings, we observed that early postnatal therapeutic-dose antibiotic treatment appears to ameliorate lipid metabolism in adult mice fed an HFD, with more pronounced effects in male mice. These improvements include reduced WAT accumulation, attenuated hepatic steatosis, and favorable blood lipid profile changes. Although some parameters did not reach statistical significance, this unanticipated effect warrants further investigation.

A limited number of studies align with our findings. Miao et al. reported that oral administration of the same antibiotic type and dose to weaned mice resulted in long-term weight reduction, but the underlying mechanism remains unclear.^22^ Similarly, Suarez-Zamorano et al. and Li et al. observed improved glucose and lipid metabolism following gut microbiota depletion in adult mice using antibiotic cocktails. We speculate that these divergent metabolic outcomes might have arisen from variations in antibiotic type, dosage, administration timing, and resultant microbial alterations. Notably, enhanced thermogenesis in iBAT or browning of ingWAT has been proposed as a potential mechanism.^49-51^ Based on these observations, we focused subsequent investigations on male mice, which exhibited more pronounced metabolic changes, with particular emphasis placed on morphological and nonshivering thermogenic functions in adipose tissues.

The developmental origin of brown adipocytes differs fundamentally from that of white adipocytes. Emerging from Myf5-expressing myogenic precursors in the paraxial mesoderm, brown adipocyte progenitors undergo lineage specification mediated by bone morphogenetic protein 7 (BMP7) and PRDM16. Subsequent terminal differentiation involves the induction of brown fat signature proteins, including UCP1, PGC-1α, CIDEA, and PPARγ. The fully differentiated state is marked by the characteristic expression of EVA1.^52^ White adipocytes originate from Myf5-negative adipogenic progenitors derived from the lateral mesoderm. Under the regulation of BMP2, BMP7, PPARγ, and CEBPs, these precursors differentiate into white preadipocytes. Sustained expression of PPARγ, CEBPs, and SREBP1 drives their terminal differentiation into white adipocytes. Alternatively, when induced by UCP1 and TMEM26, these cells can differentiate into beige adipocytes. White adipocytes can also undergo “browning” into beige-like adipocytes under specific conditions, acquiring the characteristic marker TMEM26.^52^^,^^53^ Both BAT thermogenic activation and WAT browning share common inducers, including cold exposure, endurance exercise, specific phytochemicals (e.g., capsaicin, resveratrol, caffeine), pharmacological agents (e.g., β3-AR agonists, PPARγ activators), and dietary components (e.g., omega-3 fatty acids).^23^^,^^54-57^ Emerging evidence from the past decade has revealed that the gut microbiota and microbial metabolites can directly regulate BAT thermogenesis and WAT browning ^17^^,^^58-60^ through UCP1-mediated uncoupling of oxidative phosphorylation to dissipate energy as heat, which provides metabolic benefits.

In the morphometric and molecular analyses of iBAT and ingWAT, we unexpectedly found that the HFD group did not exhibit adipocyte hypertrophy in iBAT, instead displaying signs of browning and lipid droplet miniaturization. Immunofluorescence staining and RT-qPCR further confirmed significantly elevated UCP1 expression in iBAT in HFD-fed mice. Moreover, ingWAT from these mice displayed marked upregulation of *Cidea*, *Prdm16*, and *Pparγ*, suggesting that HFD feeding activates nonshivering thermogenesis in murine adipose tissue. The existing literature predominantly indicates that chronic HFD feeding (≥8 weeks) typically suppresses BAT thermogenic capacity and lipid metabolism in mice,^26^^,^^61-63^ although some studies reported HFD-mediated stimulation of BAT activity and UCP1 expression.^64^ Notably, Ohtomo et al. revealed a time-dependent effect: short-term HFD exposure stimulated BAT thermogenesis and fatty acid oxidation, whereas thermogenic activity eventually declined to baseline levels over 20 weeks of HFD consumption.^65^ These findings suggest an initial adaptive enhancement of diet-induced thermogenesis (DIT) in BAT to counteract excessive energy intake during early HFD exposure, followed by progressive functional deterioration with prolonged fat accumulation and obesity development. DIT contributes to 5%–15% of the total daily energy expenditure through the metabolic processes of digestion and absorption. Research has demonstrated BAT’s involvement in mediating DIT,^66^^,^^67^ with additional modulation by hormonal factors such as insulin and leptin,^68^^,^^69^ consistent with our observations of HFD feeding-induced iBAT browning and elevated serum leptin levels in male mice. However, the adaptive thermogenic response and BAT activation triggered by HFD appear insufficient to prevent obesity development, suggesting that more robust BAT activation might be required for significant metabolic improvement.^70-72^

In antibiotic-exposed mice, this study revealed the link between early postnatal gut microbiota alterations and enhanced thermogenic capacity in adult iBAT without significantly affecting ingWAT. Further examination of weaning-stage iBAT development demonstrated that these early microbiota changes markedly activated mitochondrial function, brown adipocyte differentiation, and thermogenesis. This programming effect persisted into adulthood, leading to sustained enhancement of iBAT thermogenic activity upon HFD challenge that exceeded the level of DIT. These findings suggest that early postnatal antibiotic-induced gut microbiota alterations might persistently shape long-term lipid metabolism phenotypes through iBAT metabolic programming mechanisms. In traditional agricultural practice, subtherapeutic antibiotics were routinely administered to livestock to promote growth.^73^^,^^74^ Subsequent research revealed that these antibiotic-induced alterations in body weight and adiposity were mediated by gut microbiota changes rather than direct antibiotic effects.^75^ Notably, early-life antibiotic exposure was found to exert more profound effects on body composition than exposure during other developmental periods,^76^^,^^77^ consistent with the DOHaD paradigm. Interestingly, therapeutic or higher antibiotic doses often produce opposing metabolic consequences, including growth delay and resistance to diet-induced obesity in mice.^78-81^ Current evidence suggests that the divergent metabolic outcomes might depend on antibiotic dosage, class, and duration, factors that differentially reshape gut microbiota composition and potentially involve microbiota–immune crosstalk.^51^ This bidirectional metabolic regulation appears contingent on antibiotic dosing regimens and their consequent microbiota perturbations, potentially mediated through microbiota-dependent immune modulation.^51^

Research from Prof. Trajkovski’s group revealed that specific gut microbiota alterations induced by antibiotics or caloric restriction can activate type 2 immune responses, including M2 macrophage polarization and T helper cell 2 (Th2)-associated cytokine signaling, which improve glucose and lipid metabolism by promoting BAT activation and WAT browning.^49^^,^^82^^,^^83^ Additionally, multiple studies have revealed that IL-6 can induce M2 macrophage polarization and stimulate BAT activation or WAT browning, thereby reducing lipid accumulation. Notably, activated brown adipocytes can also secrete IL-6, creating an autocrine loop that further enhances thermogenesis.^84-87^ M2 macrophages express tyrosine hydroxylase, the rate-limiting enzyme in catecholamine biosynthesis, which converts tyrosine to catecholamines that bind β3-AR on adipocytes to activate thermogenic pathways.^88^^,^^89^ In this study, an M2-like phenotype (reduced *iNOS*, elevated *Arg1*) was observed in developing iBAT, coincident with increased circulating IL-6 levels but absent Th1/Th2 polarization. These findings suggest that early postnatal antibiotic exposure-associated gut microbiota alterations might be correlated with M2 macrophage polarization and IL-6 signaling, stimulating iBAT development and thermogenic programming to exert long-term effects on lipid metabolism.

This study further explored the potential gut microbiota structure metabolic programming effects. Early postnatal antibiotic exposure exerted minimal impact on gut microbiota composition and function in adult HFD-fed mice, and it did not significantly alter the HFD-induced increase in jejunal lipid absorption. This suggests that gut microbiota structure metabolic programming might not represent the major pathway by which early microbial perturbations exert lasting metabolic influences. The small intestinal microbiota plays a crucial role in regulating nutrient absorption and transport.^90^^,^^91^ Previous studies reported that germ-free mice are resistant to obesity, partly because of reduced intestinal fat digestion and absorption, characterized by low expression of fatty acid transport genes and proteins (CD36, FABP2, DGAT1, DGAT2). Conversely, conventional mice fed an HFD exhibit an altered small intestinal microbiota, leading to the upregulation of transporters to enhance lipid absorption, an effect proven to be microbiota-dependent rather than diet-mediated through fecal microbiota transplantation experiments.^73^ Consistent with these findings, our study observed upregulated jejunal lipid absorption and transport genes in HFD-fed mice independent of intestinal barrier function. These results suggest that HFD-shaped gut microbiota promotes obesity by enhancing intestinal lipid absorption, and modulating the microbiota to limit intestinal lipid absorption might represent a promising anti-obesity strategy. Future studies should aim to characterize the microbial signatures and metabolites associated with this lipid-restrictive enterotype.

Although this study demonstrated an association of early postnatal antibiotic-associated gut microbiota alterations with improved long-term lipid metabolism, these findings should not be construed as advocating antibiotic use for obesity prevention, given the observed glucose metabolic disorders in antibiotic-exposed weaning mice (Figure S1). Rather, these results suggest that strategically stimulating iBAT development through immune signaling pathways during early life might confer lasting metabolic benefits via programming effects. This warrants investigation of alternative early-life interventions with similar immunomodulatory properties (e.g., probiotic, postbiotic, omega-3 fatty acid, or breast milk component supplementation) that could promote metabolic health without adverse effects.

Several limitations of this study should be acknowledged. First, the assessment of iBAT thermogenic activation relied primarily on transcriptional profiling of key genes, supplemented by UCP1 immunofluorescence and rectal temperature measurement. Although the findings were consistent with functional improvement in lipid metabolism, more direct measures of thermogenic output, such as protein-level quantification of thermogenic markers, phosphorylation status of relevant signaling pathways, or *in vivo* thermal imaging, would strengthen the physiological relevance of these findings. Second, the proposed involvement of IL‑6 signaling and M2 macrophage polarization remains speculative. Future studies employing IL‑6 neutralization, flow cytometric analysis of macrophage subsets, spatial immunofluorescence, or conditional knockout models are needed to establish causality within this putative axis. The absence of metabolomic profiling also precluded a deeper understanding of the microbial and host metabolites that might mediate the gut–BAT axis. Third, to manage costs, fecal and blood samples were pooled, reducing the statistical power of microbiota and serum analyses. Although sample pooling is a recognized strategy in resource-limited settings, individual sample profiling would offer higher resolution and robustness. Finally, mechanistic investigations were conducted only in male mice because of their more pronounced and consistent metabolic phenotype under our experimental conditions. Consequently, whether similar mechanisms operate in female mice remains an open question. Future work should incorporate sex‑stratified designs to explore potential sex‑dependent responses in gut–adipose crosstalk. Addressing these limitations will help refine our understanding of how early‑life microbiota perturbations shape metabolic trajectories and inform the development of targeted interventions.

In summary, this study revealed that early postnatal gut microbiota exerts lasting effects on host lipid metabolism. Early postnatal antibiotic exposure-associated gut microbiota alterations were found to partially improve lipid metabolism in adult male mice, potentially mediated by BAT metabolic programming via IL-6 signaling and M2 macrophage polarization. These findings open new avenues for developing safer interventions (e.g., probiotics, postbiotics, omega-3 fatty acids) targeting this pathway during critical developmental windows, providing a novel approach to obesity prevention across the lifespan.

## Supplementary Material

Supplementary_Materials Supplementary_Materials.docx

## Data Availability

16S rRNA sequencing data reported in this study is submitted to Sequence Read Archive (SRA) under the BioProject ID PRJNA1328625 in NCBI (https://www.ncbi.nlm.nih.gov/sra/PRJNA1328625).
