# Supplementary material for: Early postnatal antibiotic-associated gut microbiota alterations might promote long-term lipid metabolism via brown adipose tissue metabolic programming
Source: Gut Microbes. 2026 May 4;18(1):2665885. doi: 10.1080/19490976.2026.2665885 (PMC13154983; doi:10.1080/19490976.2026.2665885)
Supplement: Supplementary_Materials — docx [file KGMI_A_2665885_SM8781.docx]

**Supplementary Materials**

**Table S1.** qPCR primer sequences

| Gene name | Primer | Sequence (5′-3′) |
| --- | --- | --- |
| glyceraldehyde-3-phosphate dehydrogenase (*Gapdh*) | *Gapdh*-F | GTTTGTGATGGGTGTGAACCAC |
|  | *Gapdh*-R | TCATGAGCCCTTCCACAATGC |
| myelin protein zero-like 2 (*Mpzl2*/*Eva1*) | *Eva1*-F | TGTCGTCCTACAGGTTCTGC |
|  | *Eva1*-R | ACCGTAGCAACGAAGGTAAGT |
| transmembrane protein 26 (*Tmem26*) | *Tmem26*-F | TGACGTCGAATGGCTGTCTC |
|  | *Tmem26*-R | GGTGTGCTATGCCGTTCTGT |
| uncoupling protein 1 (*Ucp1*) | *Ucp1*-F | AAAGTCCGCCTTCAGATCCAA |
|  | *Ucp1*-R | CGCTGTACAGTTTCGGCAATC |
| peroxisome proliferator activated receptor gamma (*Pparγ*) | *Pparγ*-F | TGGAGCCTAAGTTTGAGTTTGC |
|  | *Pparγ*-R | ATGTCCTCGATGGGCTTCAC |
| PPARG coactivator 1 alpha (*Pgc1α*) | *Pgc1α*-F | AACCACACCCACAGGATCAG |
|  | *Pgc1α*-R | TGAGCAGGGACGTCTTTGTG |
| cell death inducing DFFA like effector a (*Cidea*) | *Cidea*-F | ACAGAAATGGACACCGGGTAG |
|  | *Cidea*-R | GACATTGAGACAGCCGAGGA |
| PR domain containing 16 (*Prdm16*) | *Prdm16*-F | AGTCGGACAACCATGCACTT |
|  | *Prdm16*-R | TGGATCTCAGGCCGTTTGTC |
| CCAAT enhancer binding protein alpha (*Cebpα*) | *Cebpα*-F | AATGGCAGTGTGCACGTCTA |
|  | *Cebpα*-R | CCCAGCCGTTAGTGAAGAGT |
| cytochrome c oxidase subunit 8B (*Cox8β*) | *Cox8β*-F | GAAGTTCACAGTGGTTCCCAAAG |
|  | *Cox8β*-R | AGCTCTCCAAGTGGGCTAAG |
|  | *Plin1*-R | TTCGAAGGCGGGTAGAGATG |
| nuclear respiratory factor 1 (*Nrf1*) | *Nrf1*-F | ACGGCCTCATGTGTTTGAGT |
|  | *Nrf1*-R | ACTCGCGTCGTGTACTCATC |
| transcription factor A, mitochondrial (*Tfam*) | *Tfam*-F | GGGAATGTGGAGCGTGCTAAA |
|  | *Tfam*-R | ACTTCGGAATACAGACAAGACTGA |
| adrenergic receptor, beta 3 (*Adrb3*/*β3-AR*) | *β3-AR*-F | TCAACCCGGTCATCTACTGC |
|  | *β3-AR*-R | ACTCTGCCTGGCTTCAACAG |
| arginase, liver (*Arg1*) | *Arg1*-F | CTACCTGCTGGGAAGGAAGAA |
|  | *Arg1*-R | GTAAGATAGGCCTCCCAGAACC |
| nitric oxide synthase 2, inducible (*Nos2*/*iNOS*) | *iNOS*-F | GCGAAAGGTCATGGCTTCAC |
|  | *iNOS*-R | AAGGCGTAGCTGAACAAGGT |
| solute carrier family 27 (fatty acid transporter), member 4 (*Slc27a4*/*FATP4*) | *FATP4*-F | CAGTCACCCAGACAAGGGTTT |
|  | *FATP4*-R | GCCATACGATAATACCTGCTGTG |
| fatty acid binding protein 2, intestinal (*Fabp2*) | *Fabp2*-F | GCCTGGACCATTGAGGGAAAT |
|  | *Fabp2*-R | CTCCTTCATATGTGTAGGTCTGGAT |
| fatty acid binding protein 4, adipocyte (*Fabp4*) | *Fabp4*-F | CGATGAAATCACCGCAGACG |
|  | *Fabp4*-R | CCAGCTTGTCACCATCTCGT |
| CD36 molecule (*Cd36*) | *Cd36*-F | AGCCAATGCCTTTGCATCAC |
|  | *Cd36*-R | TGCCAATGTCTAGCACACCAT |
| diacylglycerol O-acyltransferase 1 (*Dgat1*) | *Dgat1*-F | TCCAGTGGGTTCCGTGTTTG |
|  | *Dgat1*-R | AGAGACAGCTTTGGCCTTGAC |
| mucin 2 (*Muc2*) | *Muc2*-F | TGCTGACGAGTGGTTGGTGAATG |
|  | *Muc2*-R | TGATGAGGTGGCAGACAGGAGAC |
| claudin-1 (*Cldn1*) | *Cldn1*-F | GCTGGGTTTCATCCTGGCTTCTC |
|  | *Cldn1*-R | CCTGAGCGGTCACGATGTTGTC |

**Table S2.** Raw Ct values of the internal reference gene in qPCR detection

| *Gapdh* Ct values in iBAT (PND28) | | | | | | | | | |
| --- | --- | --- | --- | --- | --- | --- | --- | --- | --- |
| Crtl | 21.16 | 22.18 | 21.61 | 20.91 | 20.36 | 21.44 | 20.87 | 21.24 | 20.40 |
|  | 20.55 | 20.90 | 21.46 |  |  |  |  |  |  |
| Abx | 22.30 | 21.52 | 22.41 | 21.80 | 22.08 | 22.66 | 21.37 | 20.92 | 21.50 |
|  | 21.03 | 21.53 | 21.01 |  |  |  |  |  |  |
| *Gapdh* Ct values in iBAT (PND63) | | | | | | | | | |
| ND | 22.11 | 22.61 | 22.03 | 21.69 | 22.90 | 21.61 | 21.75 | 22.71 | 21.56 |
|  | 21.32 | 21.17 | 21.95 |  |  |  |  |  |  |
| HFD | 21.96 | 21.95 | 22.17 | 22.55 | 22.56 | 22.18 | 21.95 | 22.86 | 22.15 |
|  | 22.03 | 22.62 | 22.46 |  |  |  |  |  |  |
| Abx+HFD | 21.84 | 22.58 | 21.43 | 22.16 | 21.60 | 21.91 | 22.46 | 22.31 | 21.61 |
|  | 21.64 | 21.72 | 22.03 |  |  |  |  |  |  |
| *Gapdh* Ct values in ingWAT (PND63) | | | | | | | | | |
| ND | 18.35 | 16.90 | 18.28 | 16.88 | 17.25 | 16.66 | 17.50 | 17.17 | 17.10 |
|  | 16.81 | 17.35 | 16.84 |  |  |  |  |  |  |
| HFD | 17.68 | 17.40 | 16.23 | 17.10 | 17.01 | 16.62 | 16.77 | 17.44 | 17.86 |
|  | 18.64 | 18.34 | 17.49 |  |  |  |  |  |  |
| Abx+HFD | 18.06 | 18.17 | 18.25 | 18.47 | 17.41 | 16.94 | 17.29 | 17.91 | 17.11 |
|  | 17.43 | 17.10 | 17.04 |  |  |  |  |  |  |
| *Gapdh* Ct values in proximal jejunum (PND63) | | | | | | | | | |
| ND | 17.04 | 16.70 | 16.78 | 17.18 | 17.13 | 17.10 | 17.52 | 16.39 | 16.93 |
|  | 17.32 | 17.87 | 18.03 |  |  |  |  |  |  |
| HFD | 17.57 | 18.13 | 17.65 | 17.76 | 17.19 | 17.75 | 16.81 | 17.17 | 16.97 |
|  | 17.03 | 17.43 | 16.82 |  |  |  |  |  |  |
| Abx+HFD | 16.89 | 17.13 | 16.36 | 16.90 | 17.31 | 17.91 | 17.67 | 18.10 | 17.76 |
|  | 16.99 | 17.45 | 17.34 |  |  |  |  |  |  |
| *β-actin* Ct values in iBAT (Supplemental experiment, PND21) | | | | | | | | | |
| Crtl | 26.09 | 27.82 | 25.94 | 25.60 | 25.48 | 26.55 | 26.85 | 27.33 | 26.57 |
|  | 27.12 | 27.47 | 25.36 | 27.25 | 26.29 | 27.25 |  |  |  |
| Abx | 26.17 | 26.69 | 27.72 | 27.54 | 25.50 | 28.13 | 23.69 | 25.32 | 25.05 |
|  | 24.62 | 26.16 | 25.35 | 26.99 | 26.91 | 29.23 | 27.09 | 25.57 |  |


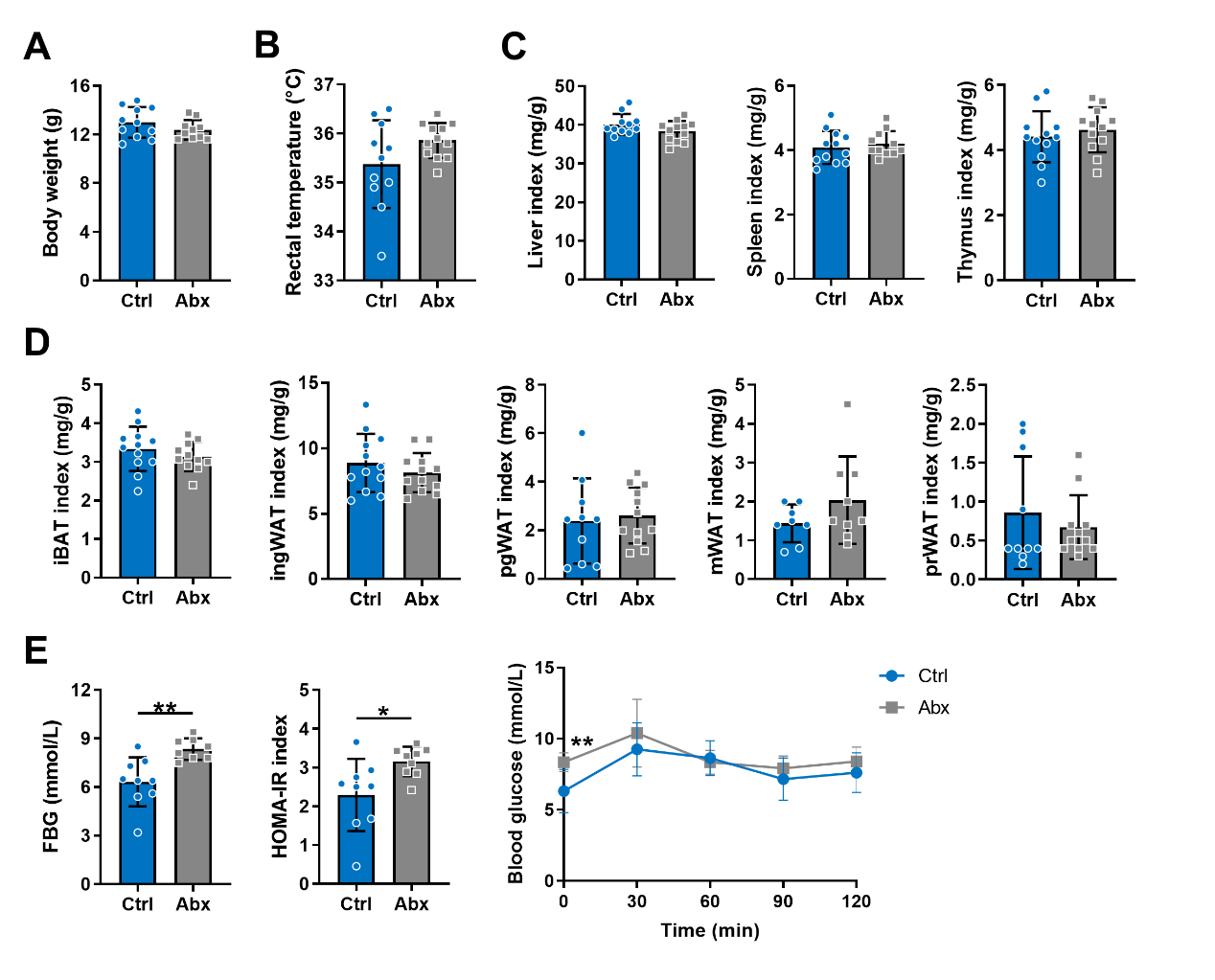


**Fig. S1. The general condition of weaning mice (PND 28).** The (A) body weight, (B) rectal temperature, (C) organ indices, and (D) adipose tissue indices of mice at PND28. (E) The glucose metabolism of weaning mice, including FBG, HOMA-IR index, and OGTT results. Data are presented as the mean ± SD (*n* = 12). ^*^*p* < 0.05, ^**^*p* < 0.01, ^***^*p* < 0.001.


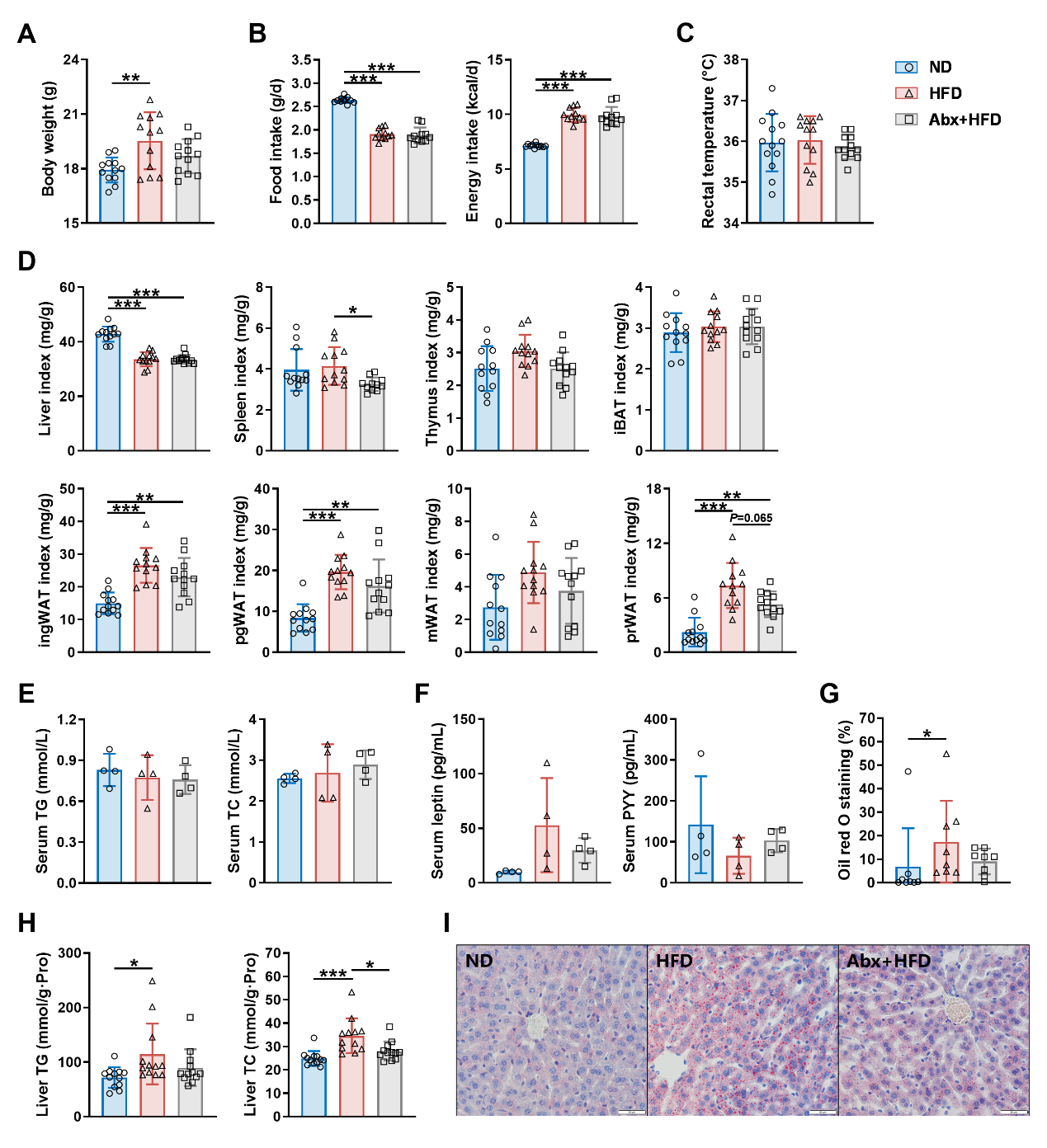


**Fig. S2. Lipid metabolic phenotypes in adult female mice (PND 63).** (A) Body weight of mice at PND63 (*n* = 12). (B) The average daily food intake and energy intake after weaning (*n* = 12). (C) Rectal temperature (*n* = 12). (D) Organ indices and adipose tissue indices (*n* = 12). (E) Serum lipid levels (*n* = 4). (F) Serum metabolic hormone levels (*n* = 4). (G) Quantification of Oil red O (*n* = 8). (H) Liver lipid levels (*n* = 12). (I) Representative images of oil red O-stained sections of liver. Scale bar, 20 μm. Data are presented as the mean ± SD. ^*^*p* < 0.05, ^**^*p* < 0.01, ^***^*p* < 0.001.


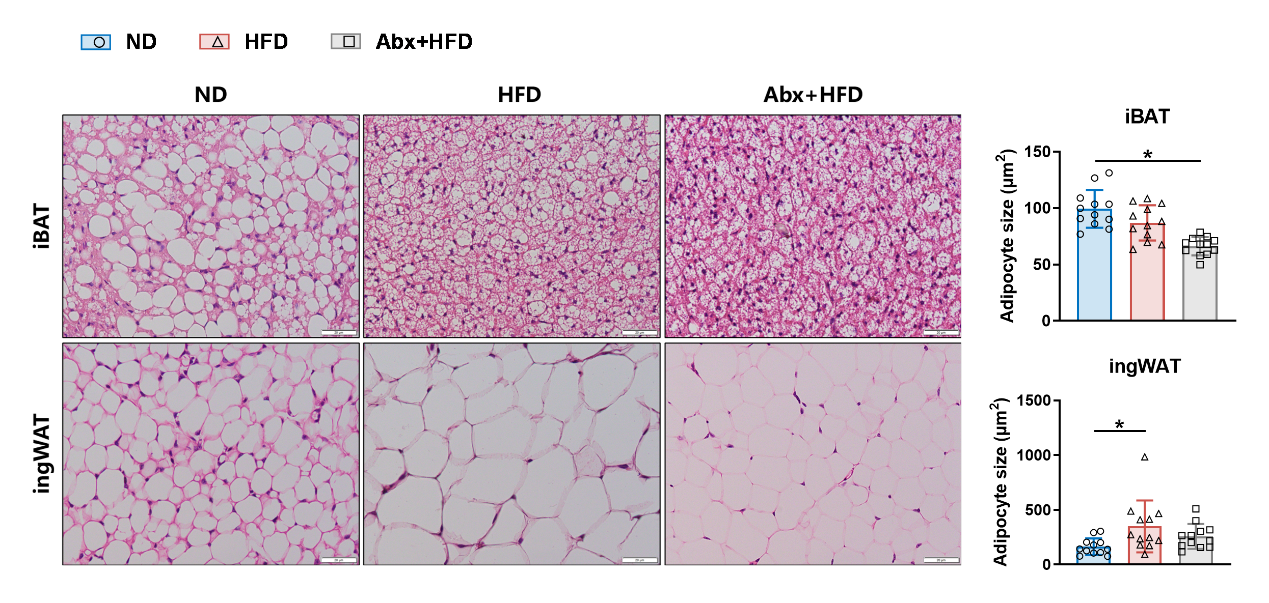


**Fig. S3. Morphological characteristics of iBAT and ingWAT of adult female mice (PND 63).** Scale bar, 20 μm. Adipocyte cross-sectional areas were quantified and compared between groups. Data are presented as the mean ± SD (*n* = 12). ^*^*p* < 0.05, ^**^*p* < 0.01, ^***^*p* < 0.001.


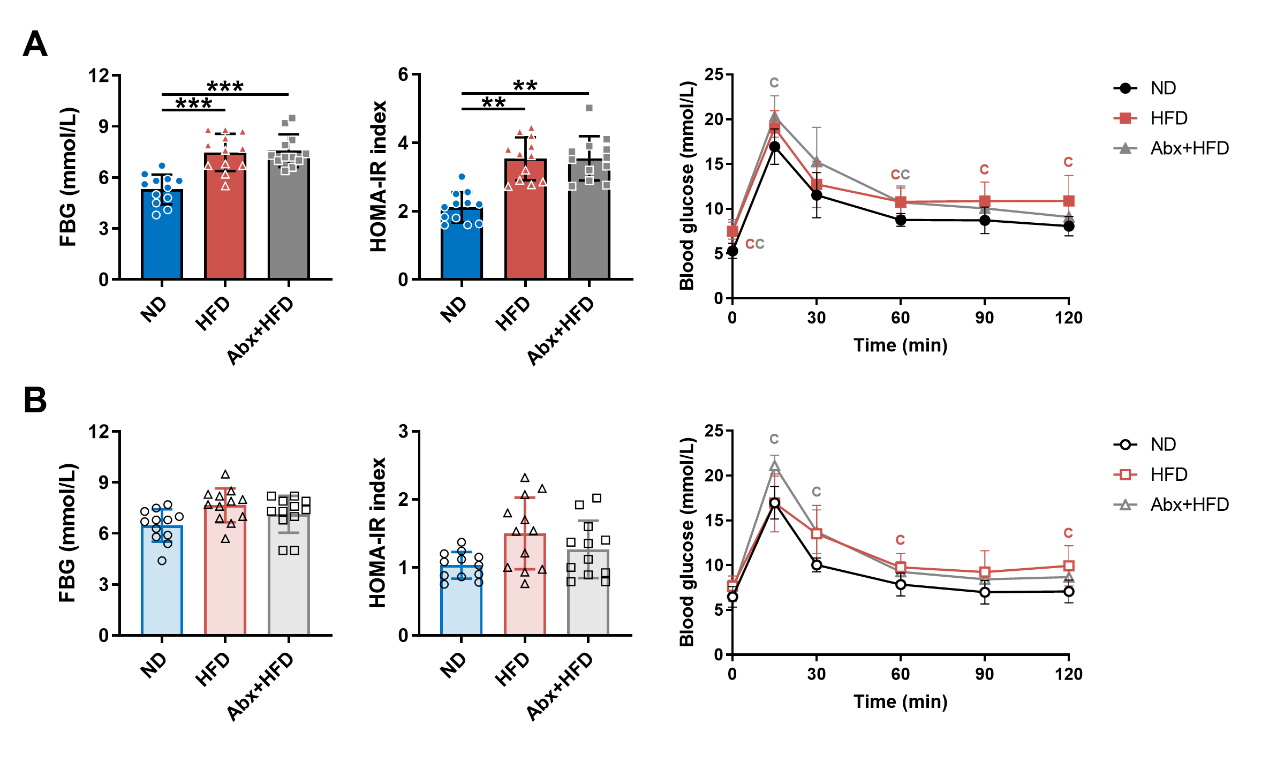


**Fig. S4. The glucose metabolism of adult mice (PND 63).** (A) The glucose metabolism of male mice. (B) The glucose metabolism of female mice. Data are presented as the mean ± SD (*n* = 12). Symbols mark statistically significant comparisons (*P* < 0.05): red "C" (HFD vs. Ctrl), gray "C" (Abx+HFD vs. Ctrl). ^*^*p* < 0.05, ^**^*p* < 0.01, ^***^*p* < 0.001.
